# Chronic tubercular thoracic empyema

**DOI:** 10.11604/pamj.2025.51.3.47431

**Published:** 2025-05-06

**Authors:** Ashwin Karnan

**Affiliations:** 1 Department of Respiratory Medicine, Datta Meghe Institute of Higher Education and Research, Sawangi (Meghe), Wardha, Maharashtra, India

**Keywords:** Tuberculosis, parapneumonic effusion, fibrothorax, thoracoscopy

## Image in medicine

A 46-year-old female, a farmer, presented with complaints of fever, right-sided chest pain, breathlessness, loss of weight, and loss of appetite for the past 3 months. She had tubercular pleural effusion 3 years back, for which she only took treatment for 2 months. A chest X-ray and high-resolution computed tomography of the patient’s thorax showed loculated right pleural effusion with thickening of the pleura with bilateral pulmonary cysts and centrilobular nodules in the tree-in-bud pattern. The patient was taken up for thoracoscopy. loculations were broken, pleural fluid drained, and multiple pleural biopsies were taken. Histopathological examination showed multinucleated giant cells and central caseating necrosis surrounded by epithelioid histiocytes, confirming the diagnosis of tuberculosis. The patient was started on antitubercular therapy and is currently on follow-up. Empyema is defined as the presence of pus in the pleural cavity. Alcoholism, intravenous drug abuse, poor oral hygiene, diabetes, and liver cirrhosis are the common risk factors. It has 3 stages: exudative stage, fibro-purulent stage, and chronic organizing stage. Computed tomography scan is the most definitive study, with the hallmark sign being the split pleura sign. Diagnostic and therapeutic modalities include thoracocentesis, tube thoracostomy and intrapleural fibrinolytics, video-assisted thoracoscopic surgery, and medical thoracoscopy. The prognosis is poor if not treated appropriately, with 1-year mortality around 20%. Treatment depends upon the causative organism, which could be tubercular, community-acquired, or hospital-acquired infection. Complications include fibrothorax, bronchopleural fistula, sepsis, empyema necessitans, and respiratory failure.

**Figure 1:**
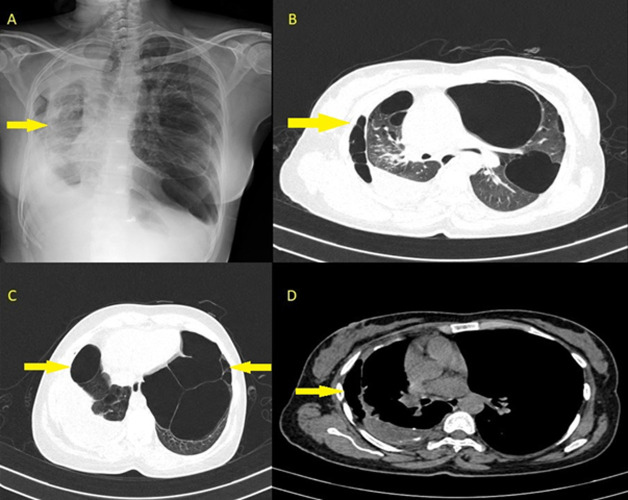
A) chest X-ray of the patient; high-resolution computed tomography of the patient’s thorax showing: B) pleural effusion with multiple air foci within the pleura; C) multiple bullae; D) thickened visceral and parietal pleura

